# BENEFITS OF A CLINICAL PATHWAY IN TOTAL KNEE ARTHROPLASTY

**DOI:** 10.1590/1413-785220243201e269506

**Published:** 2024-03-22

**Authors:** Márcio de Castro Ferreira, Gilvânia Silva, Carolina Padrão Amorim Marinelli, Julia Souza de Oliveira, Pedro Aurélio Mathiasi, Gilberto Luis Camanho

**Affiliations:** 1 HCor, São Paulo, SP, Brazil.

**Keywords:** Arthroplasty, Replacement, Knee, Managed Care Programs, Quality of Health Care, Artroplastia do Joelho, Programas de Assistência Gerenciada, Qualidade da Assistência à Saúde

## Abstract

**Objective:**

Demonstrate whether a multiprofessional Clinical Pathway Program in Total Knee Arthroplasty (CPPA) contributesto optimizing hospital care.

**Method:**

Retrospective study of medical data of care indicators in 310 patients divided into two groups: A- who underwent arthroplasty in the last biennium before the introduction of the CPPA (n=144) and group B- who underwent TKA in the biennium after the introduction of the CPPA (n=166).

**Results:**

Postoperative showed a significant difference in favor of group B over group A for hospitalization time in days 4.33 ± 2.79 and 5.4 ± 1.67 (p<0.001), time of prophylactic antibiotic in hours 28.13 ± 33.77 and 81.49 ± 40.91 (p<0.001), referral to the intensive care unit 40.9% and 73.4% (p<0.001), initiation of thromboprophylaxis within 24 hours 97.9% and 82.5% (p<0.001), use of elastic stockings and/or intermittent compression prescribed for thromboprophylaxis 89.5% and 31.2% (p<0.001), initiation of rehabilitation within 24 hours 90.1% and 66.1% (p<0.001), readmissions within 30 days 4.1% and 3% (p = 0.76), readmissions 90 days 2.7% and 6.6% (p = 0.183), transfusions 5.5% and 15.2% (p = 0.033).

**Conclusion:**

The implementation of a multiprofessional CPPA contributed to the implementation of care protocols, favoring greater patient safety. ***Level of Evidence III; Retrospective Comparative Study.***

## RESUMO

**Objetivo:**

Demonstrar se um Programa de Cuidados Clínicos multiprofissional em Artroplastia Total de Joelho (PCCA) contribui para a otimização assistencial hospitalar.

**Método:**

Estudo retrospectivo em prontuários de indicadores assistenciais em 310 pacientes divididos em dois grupos: A- submetidos a artroplastia no último biênio antecessor a introdução do PCCA (n=144) e grupo B- submetidos a ATJ no biênio após a introdução do PCCA (n=166).

**Resultados:**

Indicadores pós-operatórios mostraram diferença significativa a favor do grupo B sobre o grupo A para tempo de hospitalização em dias 4,33 ± 2,79 e 5,4 ± 1,67 (p<0,001), tempo de antibiótico profilático em horas 28,13 ± 33,77 e 81,49 ± 40,91 (p<0,001), encaminhamento para unidade de terapia intensiva 40,9% e 73,4% (p<0,001), início da tromboprofilaxia dentro de 24h 97,9% e 82,5% (p<0,001), uso de meias elásticas e/ou compressão intermitente prescritos para tromboprofilaxia 89,5% e 31,2% (p<0,001), tempo para iniciação da reabilitação em 24h 90,1% e 66,1% (p<0,001), readmissões em 30 dias 4,1% e 3% (p = 0,76), readmissões 90 dias 2,7% e 6,6% (p = 0,183), transfusões 5,5% e 15,2% (p = 0,033).

**Conclusão:**

A implementação de um PCCA multiprofissional contribuiu para o cumprimento dos protocolos assistenciais favorecendo maior segurança para os pacientes. ***Nível de Evidência III; Estudo Retrospectivo Comparativo.***

## INTRODUCTION

Total knee arthroplasty (TKA) is the gold standard therapeutic procedure and widely performed for the treatment of gonarthrosis.^1-3^ It is noteworthy the exponent growth of this surgery, which is accompanied by an increase in clinical complications associated with large additions of financial resources, a fact that adds relevance to the care issue in order to mitigate the risks involved in these surgeries.^2-7^

In this context, in order to amplify the therapeutic standardization and optimization of the best medical practices, the Clinical Pathway Programs in Arthroplasties (CPPA) in which multiprofessional teams are trained and monitored to execute institutional care protocols are presented as an alternative for hospital management.^1,8,9^

A CPPA aims to guide, monitor and generate action plans for health care based on scientific evidence within the culture of a health institution.

The objective of this study is to assess whether the implementation of a CPPA under the guidance of the Joint Commission International (JCI) can increase the safety of care for patients undergoing total knee arthroplasty.

## MATERIALS AND METHOD

The retrospective study of patients records analysis was carried out at our Hospital after approval by the Research Ethics Committee (number CAAE 61212716.0.0000.0060). The patients were divided into two groups: Group A (GA) patients who underwent TKA in the last biennium prior to the introduction of CPPA (2013-2014), and Group B (GB) patients who underwent TKA in the last biennium after the start of the CPPA without the impact of the Covid-19 pandemic (2018-2019).

The care indicators identified in the medical records were established and required by the JCI for the CPPA, as follows:

- length of hospital stay (LOS);
- administration of antibiotic prophylaxis (ATBp) – interval 60min to 05min before the start of surgery;
- suspension of ATBp within 24 hours;
- average ATBp administration time;
- initiation of prophylaxis for deep vein thrombosis (DVT) within 24 hours postoperatively;
- use of adjuvant methods for prophylaxis DVT– intermittent pneumatic compression device (IPCD) and/or compression stockings (CS);
- immediate postoperative care location: Intensive Care Unit (ICU) or Inpatient Unit Care (IUC);
- time to start postoperative physical therapy rehabilitation;
- time to start the postoperative gait;
- readmissions 30 days after surgery;
- readmissions at 90 postoperative days;
- blood transfusion;

All indicators were identified by a single researcher with absolute anonymity of the samples, thus excluding the need to apply the written informed consent form of the participants.

In addition to the care indicators presented, the population profile was also compared between the groups through: age, sex, laterality, body mass index (BMI) and Physical State Classification of the American Society of Anesthesiologists (ASA).

All patients who underwent primary unilateral TKA were included in the study. Patients in both groups whose medical records did not contain all the information for the indicators defined for the study were excluded.

### Statistical analysis

The indicators referring to the patients as well as the care performances of the CPPA were presented through categorical variables described in absolute and relative frequencies, and through numerical variables, described by the mean, standard deviation, median and quartiles. For the comparative statistical analysis between the data obtained from the studied groups, the Fisher’s Exact test and the Mann-Whitney test were used.

The tests used a significance level of 5% and the analyzes were carried out with the help of the statistical software R (R Core Team-2021).

## RESULTS

A total of 168 patients were identified in group A (GA), two of which were excluded, resulting in a final sample of 166 patients. In group B (GB), 146 patients were identified, two of which were excluded, resulting in 144 patients, totaling 310 patients evaluated.

The comparative population analysis between the groups showed no statistically significant difference for age (p = 0.896), sex (p = 0.529), laterality (p = 0.374), BMI (p = 0.881) and ASA (p = 0.076). Female patients were more prevalent in both groups (74% in GA and 69% in GB). The mean age, BMI and ASA were 69 years old, 29.8 Kg/m2 and 2 in AG, and 70 years old, 29.8 Kg/m2 and 2 in GB, as shown in Table 1.

**Table 1:** Indicators and comparative statistical analysis of population epidemiological data from groups A (GA) and B (GB). Body Mass Index (BMI), American Society of Anesthesiologists Physical Status Classification (ASA), Total Knee Arthroplasty (TKA).

	Before CPPA (n=166)	After CPPA (n=144)	Total (n=310)	P
Age average	69.65± 8.67	70.28± 7.59	69.94± 8.18	0,896
Age median	70.5	70	70	
**Sex**				**0,529**
Male	45/166 (27.11%)	45/144 (30.77%)	90/310 (28.8%)	
Female	121/166 (72.89%)	99/144 (69.23%)	220/310 (71.2%)	
**Laterality**				**0,374**
TKA right	94/166 (56.63%)	75/144 (52%)	169/310 (54,5%)	
TKA left	72/166 (43.37%)	69/144 (48%)	141/310 (45,5%)	
**ASA**				**0,076**
1	12/166 (7.23%)	3/144 (2.08%)	15/310 (4.84%)	
2	139/166 (83.73%)	131/144 (90.97%)	270/310 (87.1%)	
3	15/166 (9.04%)	10/144 (6.94%)	25/310 (8.06%)	
BMI average	29.86 ± 4.75	30.02± 4.27	29.93± 4.53	0,881
BMI median	29.5	29.18	29.36	

Regarding the management of preoperative ATBp induction, it was not statistically different (p = 0.259) since 97.5% of the GA patients and 97.7% of the GB patients received the medication within the time interval between 60 and 5 minutes prior to the surgical incision, with cefuroxime being the most widely administered medication. Regarding the extension period of ATBp, approximately 2% of the patients in the GA and 91% of the patients in the GB discontinued the use of antibiotics within 24 hours after the end of the surgery (p < 0.001). The mean post-surgical ATBp extension time was 81.4h in GA and 28h in GB, with statistical significance of p < 0.001, as shown in Table 2.

**Table 2:** Indicators and comparative statistical analysis of data associated with prophylactic antibiotic therapy and thromboprophylaxis (ATBp) in groups A (GA) and B (GB). Antibiotic (BMI), deep vein thrombosis (DVT), postoperative (PO), intermittent pneumatic compression device (IPCD).

	Before CPPA (n=166)	After CPPA (n=144)	Total (n=310)	P
**management ATB 60 - 5 min before surgery**				**0,259**
No	4/166 (2,4%)	3/144 (2.1%)	7/310 (2,3%)	
Yes	162/166 (97,5%)	141/144 (97.9%)	303/310 (97,7%)	
ATBp (hours); average	81.49 ± 40.91	28.13 ± 33.77	56.79 ± 46.18	<0,001
**ATBp suspension within 24h**				**<0,001**
No	163/166 (98.19%)	15/144 (10.42%)	178/310 (57.42%)	
Yes	3/166 (1.81%)	129/144 (89.58%)	132/310 (42.58%)	
**Prophylaxis DVT within 24h**				**<0,001**
No	29/166 (17.47%)	3/144 (2.08%)	32/310 (10.32%)	
Yes	137/166 (82.53%)	141/144 (97.92%)	278/310 (89.68%)	
**Mechanical prophylaxis**				**<0,001**
compression stockings	28/166 (16.87%)	72/144 (50%)	100/310 (32.26%)	
compression stockings + IPCD	24/166 (14.46%)	57/144 (39.58%)	81/310 (26.13%)	

For the pharmacological thromboprophylaxis indicator started within 24 hours of the end of surgery, a significant improvement was observed in GB (p = <0.001), with enoxaparin being the most used drug in both groups. It was observed that in GB the association of adjuvant mechanical methods in the lower limbs was present in 31.2% of the GA and in 89.5% of the GB (p < 0.001). (Table 2)

The beginning of physical therapy rehabilitation showed results with a wide care difference in relation to the groups, since in the GA 66.1% of the patients started rehabilitation activities on the first postoperative day while in the GB 90.1% received assistance in the same period (p. < 0.01). The beginning of gait also showed positive results, as in the GA only 7% of the patients walked on the first postoperative day, while in the GB 63.8% performed gait training in the same period (p < 0.01). (Table 3)

**Table 3:** Indicators and comparative statistical analysis of data associated with physical therapy rehabilitation postoperative period (PO) and blood transfusion in groups A (GA) and B (GB).

	Before CPPA (n=166)	After CPPA (n=144)	Total (n=310)	P
**Physiotherapy**				
Immediate PO	63/166 (37.95%)	75/144 (51.75%)	138/310 (44.5%)	<0,001
1º PO	47/166 (28.31%)	55/144 (38.46%)	102/310 (32,9%)	
2º PO	46/166 (27.71%)	14/144 (9.79%)	60/310 (19.3%)	
3º PO	8/166 (4.82%)	0/144 (0%)	8/310 (2.5%)	
4º PO	2/166 (1.2%)	0/144 (0%)	2/310 (0.6%)	
**Walk**				
Immediate PO	0/166 (0%)	13/144 (9.03%)	13/310 (4.19%)	<0,001
1º PO	12/166 (7.23%)	79/144 (54.86%)	91/310 (29.35%)	
2º PO	95/166 (57.23%)	51/144 (35.42%)	146/310 (47.1%)	
3º PO	49/166 (29.52%)	1/144 (0.69%)	50/310 (16.13%)	
4º PO	7/166 (4.22%)	0/144 (0%)	7/310 (2.26%)	
6º PO	3/166 (1.81%)	0/144 (0%)	3/310 (0.97%)	
**Readmission within 30 days**				
No	161/166 (96.99%)	138/144 (95.83%)	299/310 (96.45%)	0,76
Yes	5/166 (3.01%)	6/144 (4.17%)	11/310 (3.55%)	
**Readmission within 90 days**				
No	155/166 (93.37%)	140/144 (97.22%)	295/310 (95.16%)	0,183
Yes	11/166 (6.63%)	4/144 (2.78%)	15/310 (4.84%)	
**Blood transfusion**				**0,033**
No	144/166 (86.75%)	136/144 (94.44%)	280/310 (90.32%)	
Yes	22/166 (13.25%)	8/144 (5.56%)	30/310 (9.68%)	

The percentages of readmissions at 30 and 90 days did not show significant statistical differences (p = 0.76 and p = 0.183), however, it was observed in the GA that 9.6% of patients (01 cellulitis, 01 wound debridement, 01 debridement surgery, 01 extensor mechanism rupture, 03 suspected DVT, 07 arthrofibrosis, 01 urinary infection, 01 patellar dislocation) and in GB 6.9% of patients (01 transient ischemic, 01 dehydration, 01 urinary infection, 02 arthrofibrosis, 01 drainage hematoma, 02 incision debridement, 01 surgical debridement, 01 erysipelas) required hospital care after surgical discharge. (Table 3)

The number of blood transfusions was significantly different between the groups (p = 0.033). In GA 15.2% of patients and in GB and 5.5% in GB required this therapy. (Table 3)

Regarding the place of hospital stay in the immediate postoperative period, it was observed that in the GA only 26.5% of the patients were referred to the UI, while in the GB the percentage of this assistance was 59%, exposing a significant behavioral change in care for not using the ICU for postoperative support (p < 0.001). The mean length of hospital stay (LOS) for GA patients was 5.4 days and 4.3 days for GB patients, showing that CPPA significantly contributed to the decrease in LOS (p < 0.001). (Table 4)

**Table 4:** Indicators and comparative statistical analysis of data associated with the patient’s place of stay in the postoperative period (PO) whether in the inpatient unit care (ICU) or intensive care unit (ICU) and the length of hospital stay in the groups A (GA) and B (GB).

	Before CPPA (n=166)	After CPPA (n=144)	Total (n=310)	P
**Immediate postoperative care**				**<0,001**
IUC	44/166 (26.51%)	85/144 (59.03%)	129/310 (41.61%)	
ICU	122/166 (73.49%)	59/144 (40.97%)	181/310 (58.39%)	
Lenght of stay; average	5.4± 1.67	4.33± 2.79	4.9± 2.32	<0,001
Lenght of stay; median	5	3	5	

## DISCUSSION

The results found in the study showed that a Clinical Care Program in Arthroplasty can contribute to the gain of hospital care performance, positively impacting the response of protocols and institutional guidelines.

The essence of a CPPA is to act in the control and compliance of the best therapeutic practices, intervening with medical professionals, physiotherapists, pharmacists, psychologists and nursing staff in constructive tasks that generate controls and feedback on the performance of their actions in the therapeutic management of patients and, thus, engaging professionals in a process of continuous improvement, reducing the risk of complications and waste of resources.^3,10-15^

Regarding the prescription of pATB during TKA, although drug induction did not present a significant difference between the groups, a disruptive change was observed associated with the extension of postoperative administration with the introduction of CPPA (p < 0.001) with the suspension in 24h. This behavioral change is very relevant for the sustainability of public health since Mobarki et al^16^ and Chokshi et al^17^ expressed the worrying global crisis that we are experiencing due to antibiotic resistance due to the misuse of these drugs.^18^ Although there is scientific discussion regarding the extent of pTAB management in arthroplasties, it is currently defined by consensus and international guidelines that the 24-hour period would be ideal if there is no clinical peculiarity that requires another care profile.^19^

The prescription of thromboprophylaxis was also largely optimized with the implementation of CPPA not only in the pharmacological form, but also with the association of adjuvant therapy such as intermittent compression devices and/or elastic compression stockings, which showed a significant increase in use (p < 0.001) without impairing the beginning of patients’ physical mobilization and rehabilitation activities.^20-22^

Mosaad et al^23^ and Weng et al^24^ described that the pharmacological routine for thromboprophylaxis is relevant and a priority, however the time of drug administration is essential to add prophylactic efficiency and, in this context, a significant improvement in the drug administration within 24 hours of surgery, indicating that patients were in better compliance with the institutional protocol to reduce the risk of thromboembolism.

A relevant point is the management of general care associated with the immediate postoperative follow-up. In the case of elderly patients and often linked to comorbidities under the care responsibility of an orthopedic surgeon, it is understandable that the medical team is insecure in the face of potential clinical instabilities that may occur in patients resulting from organic responses to surgical trauma. This context enhances the referral of patients to the ICU after surgery, a place that empirically increases the risks in agreement with Barnett et al^25^ Despotovic et al.^26^

One of the contributions of the CPPA to mitigate the use of the ICU in the immediate postoperative period was the establishment of a standardization of preoperative surgical risk screening performed by a team of cardiologists who monitored the daily evolution of patients during the perioperative care journey together with the surgical team providing the patient with a double medical guardianship during the hospital stay.

It would be opportune to amplify, in Brazilian private hospitals, intermediary care support in the immediate postoperative period in order to reduce the need to refer some patients to intensive care units that require more attention. Our institution, at the moment, does not have an intermediate level between the ordinary inpatient unit and intensive care.

In relation to blood transfusions Slover et al^27^ demonstrated that this need can reach 8% in arthroplasties. This study showed a positive response related to this indicator in the post-CPPA group with a decrease of 7.7%. A relevant factor for this finding is due to the better control and clinical stabilization of anemias during the preoperative evaluation, in addition to the establishment of criteria for the indications of transfusions that are shared with the clinical team and not only the judgment of the orthopedic surgeon.

Physiotherapy rehabilitation protocols focusing on the "fast track" concept were created by physical therapists and agreed with orthopedic surgeons in order to optimize recovery during hospital stay. This action significantly reflected in the improvement in the beginning of physical mobility and gait in the immediate postoperative period and on the first postoperative day, with a percentage of 90.1% of patients in gait activity within 24 hours of the postoperative period. This condition certainly had an impact on reducing the length of hospital stay, corroborating the results demonstrated by Foni et al,^3^ Tayrose et al^28^ Ayalon et al.^29^

It was observed that readmissions for arthrofibrosis decreased in patients operated after CPPA and we postulate that this condition may be linked to rehabilitation started earlier, as well as the sharing of a booklet with exercises that patients should perform daily at home for the functional optimization of the operated knee.

All these care optimizations allowed patients undergoing surgery after CPPA to reach the clinical and orthopedic criteria for hospital discharge established by the presence of walking with the aid of walkers, controlled and tolerable pain with oral medications, and range of motion of the operated knee at 90º in length of stay 20% shorter than pre-PCCA patients, without increasing readmission rates.

### Limitations of the study

As in any retrospective study of data analysis in medical records, the number of patients evaluated and the quality of the data identified in the records can always carry empirical bias for the results found.
